# Gender Bias Impacts Top-Merited Candidates

**DOI:** 10.3389/frma.2021.594424

**Published:** 2021-05-10

**Authors:** Emma Rachel Andersson, Carolina E. Hagberg, Sara Hägg

**Affiliations:** ^1^Department of Biosciences and Nutrition, Karolinska Institutet, Stockholm, Sweden; ^2^Department of Cell and Molecular Biology, Karolinska Institutet, Stockholm, Sweden; ^3^Division of Cardiovascular Medicine, Department of Medicine Solna, Center for Molecular Medicine, Karolinska Institutet and Karolinska University Hospital, Stockholm, Sweden; ^4^Department of Medical Epidemiology and Biostatistics, Karolinska Institutet, Stockholm, Sweden

**Keywords:** diversity, life science, peer review, bibliometry, faculty positions, gender, equality

## Abstract

Expectations of fair competition underlie the assumption that academia is a meritocracy. However, bias may reinforce gender inequality in peer review processes, unfairly eliminating outstanding individuals. Here, we ask whether applicant gender biases peer review in a country top ranked for gender equality. We analyzed peer review assessments for recruitment grants at a Swedish medical university, Karolinska Institutet (KI), during four consecutive years (2014–2017) for Assistant Professor (*n* = 207) and Senior Researcher (*n* = 153). We derived a composite bibliometric score to quantify applicant productivity and compared this score with subjective external (non-KI) peer reviewer scores of applicants' merits to test their association for men and women, separately. To determine whether there was gender segregation in research fields, we analyzed publication list MeSH terms, for men and women, and analyzed their overlap. There was no gendered MeSH topic segregation, yet men and women with equal merits are scored unequally by reviewers. Men receive external reviewer scores resulting in stronger associations (steeper slopes) between computed productivity and subjective external reviewer scores, meaning that peer reviewers “reward” men's productivity with proportional merit scores. However, women applying for assistant professor or senior researcher receive only 32 or 92% of the score men receive, respectively, for each additional composite bibliometric score point. As productivity increases, the differences in merit scores between men and women increases. Accumulating gender bias is thus quantifiable and impacts the highest tier of competition, the pool from which successful candidates are ultimately chosen. Track record can be computed, and granting organizations could therefore implement a computed track record as quality control to assess whether bias affects reviewer assessments.

## Introduction

Fostering groundbreaking research requires identification of the best ideas and individuals. Competition in academia is fierce, and only 47% of those that are awarded a PhD pursue a career within science after their PhD, and only 0.45% become a professor (The Royal Society, 2010). Resources to support researchers are limited, and thus, it is essential that the best candidates be identified, in order to use resources wisely. However, bias may limit career progression for women or minorities. Recent data suggest that both overt and implicit bias is generally decreasing across a majority of social-group attitudes including sexual orientation, race, skin tone, age, disability, and body weight (Charlesworth and Banaji, 2019), yet women still make up only one third of all professors in European countries (Swedish Higher Education Authority, 2018; European Commission, 2019; Hovdhaugen and Gunnes, 2019).

Bias can affect multiple aspects of a scientific career, including decision making in recruitment (Steinpreis et al., 1999; Moss-Racusin et al., 2012), grant awarding (Wennerås and Wold, 1997; Holst and Hägg, 2018; Witteman et al., 2019), and citations (Caplar et al., 2017). A common concept is that a portion of the differences in success rates between men and women can be attributed not to bias, but rather to boys/men and girls/women choosing different fields within STEM, wherein men enter more competitive highly cited fields, while women enter less competitive research fields (Riegle-Crumb et al., 2019; Buser et al., n.d.). However, girls/women, including highly gifted girls (Lubinski et al., 2001), tend to choose medicine/biology more often than men (Cheryan et al., 2016; Riegle-Crumb et al., 2019), and it is therefore of interest to assess whether and how bias may impact on career progression within medicine and biology, which initially attract more women than men. An entry-level group leader position in academia is typically awarded following peer review of the applicants and their proposed projects, assessing quality, innovativeness, feasibility, and potential. This review process can be assisted by “objective” quantification of merits, for example, bibliometry. While simple bibliometry is associated with numerous pitfalls (Belter, 2015), composite bibliometry that takes into account multiple bibliometric parameters is a powerful tool that has previously been used to objectively measure productivity (Holst and Hägg, 2018) and is a strong predictor of scientific quality, for example, more accurately identifying Nobel Prize winners than citations alone (Ioannidis et al., 2016).

In order to assess whether, and how, bias impacts upon career progression, we ask whether reviewer assessments of men and women candidates correlate with their objective merits. By introducing a quantification of actual merits, rather than simply analyzing rates of success, we test whether candidates are correctly recognized for their merits, as a factor of gender. In order to test whether applicants are suitably rewarded for their merits, we analyzed the peer-review procedure for positions awarded at a Swedish medical university, Karolinska Institutet (KI), over a period of 4 years, including 1,187 eligible applicants, of which 30% (360 applicants) were selected for peer review by external non-KI reviewers in Sweden. “Public access to information” (=Offentlighetsprincipen) is mandated in Swedish law, allowing insight into the activity of government organizations and providing here a unique transparent insight into a real-world recruitment procedure. By computing a composite bibliometric score, which includes seven publication parameters, as a proxy for productivity, we quantify the applicants' objective records of accomplishment. The composite bibliometric score was compared with the external reviewer scores, to assess whether men and women of equal productivity were considered equally merited. In addition, we examine whether men and women applicants represent gender-segregated subfields within biology and medicine. Our results show that men are generally awarded higher merit scores by reviewers for equal productivity, and we show that this discrepancy in scoring is greatest at the top of the competition, and not reflected by subfield segregation. While much has improved with regard to equal opportunities, continued efforts to eliminate bias are clearly needed to achieve parity, in particular, in assessing top-ranked candidates.

## Methods

### Data

The academic trajectory at KI is, in general, PhD studies (4 years), postdoctoral studies (5 years), Assistant Professor (independent entry-level group leader position after postdoc, 4 years that can be extended to a total of 6 years), followed by Senior Researcher (Associate Professor equivalent, 5 years), and Professor. In the time-span covered by this analysis, there was no tenure track or promotion between positions. As there is no promotion from Associate Professor to Professor, it is possible to remain in the Associate Professor/Senior Researcher stage indefinitely as long as the PI attracts funding. Each year since 2014, KI has announced position grants to recruit Assistant Professors and Senior Researchers within a Career Ladder scheme (https://ki.se/en/about/faculty-funded-career-positions). This program entails a three-step process, in which applicants submit a CV and a project plan. In Step 1, the top 30% of the applicants are selected for external peer review. In Step 2, these top applications are assessed by external reviewers. Six external reviewers, professors at other Swedish universities, with equal gender distribution, score each application according to instructions provided by KI. They provide one score for merits (based on the CV) and one for the project plan, returning a ranking to KI. The merit scores, analyzed in this study, are based on (1) publications and (2) academic education and research merits. Publications include published papers and scientific presentations. Academic education and research merits includes education, experience of research, competence, independence, research network, financing, prizes, and invitations to present or review. Applicants are graded between 0 and 7, where 7 = Exceptional, 6 = Excellent, 5 = Very good to Excellent, 4 = Very Good, 3 = Good, 2 = Weak, 1 = Poor, 0 = Insufficient information provided for assessment. To test whether different reviewers use different scales in assigning scores, and whether data needed to be normalized or anchored, the KI Library normalized reviewer scores using Z-normalization and tested how the normalization impacted the scores. Z-normalization compensates for different grading scales between assessors if these exist. For Z-normalization, the difference between an individual grade and the mean value of the assessor's grade is divided by the standard deviation of the assessor's grade: Z = (X – M)/S. Intraclass correlation coefficients before and after standardization were determined using the package “psych” in R: greater homogeneity in the data result in larger coefficients. The intraclass correlation coefficients were sufficiently high in 2014, 2015, and 2016 (not tested in 2017) that this normalization was not deemed necessary from 2017 onward. All analyses are performed on the non-normalized data. In Step 3, the top 20 or so applicants are interviewed by KI professors, competing for one of 7–11 positions for Assistant Professor, or one of 6–8 positions as Senior Researcher. There is no selection based on research field or type of research.

The data in this study include information on applications for KI Career Ladder positions between 2014 and 2017, total number of eligible applicants, number sent to external review, number that went to interview, and number awarded. “Public access to information” (=Offentlighetsprincipen) is a fundament of Swedish law, granting access to this type of information. However, in order to protect individuals' integrity, data are presented on a group level only, precluding identification of individuals. All applications that were sent to external review were included in the following analysis. In total, 360 applications from Step 2 (external review) were assessed, of which 207 were applications for Assistant Professor and 153 for Senior Researcher. The data included gender and the average external non-KI reviewer scores of the applicant's merits. Data were provided by the KI registrar.

The 2014 data for Assistant Professors have been analyzed and reported once before using a slightly different composite bibliometric score (Holst and Hägg, 2018).

The data for year 2015 deviate from other years, with a sharp increase in the number of applications to both Assistant Professor positions and Senior Researcher positions. The deviation is explained by a change in the eligibility criteria in 2015, now allowing applications from persons holding a permanent position (as lab manager, project leader, or similar), while these applicants had been excluded (ineligible) in 2014.

### Medical Subject Heading Term Analysis

For the same articles analyzed in the composite bibliometric score, the associated MeSH terms (Medical Subject Headings) were collected, and only terms described as “major descriptor” in PubMed were used. For each person, if at least two articles had the same MeSH term, the applicant was linked to that specific term. To maintain anonymization of applicants, for a MeSH term (node) to show up in the network figure, at least two Assistant Professor applicants or three Senior Researcher applicants had to have the same term. If a person has more than one MeSH term (node) in the network, an edge is created between them. The MeSH term network figures were created by men and women separately to highlight the gender differences. The network data files were created in R and the network figures were done in Gephi with the Force Atlas option.

To analyze the overlap between the research fields of applicants of either gender, the MeSH Browser (https://meshb.nlm.nih.gov/) was used to retrieve the parental MeSH categories (meaning the highest category in the hierarchical MeSH tree) for each gender using their respective top 50 MeSH terms (nodes). MeSH term analysis was done separately for Assistant Professor applicants and Senior Researcher applicants, yielding a list 28 parental categories for Assistant Professor applicants and 21 for Senior Researcher applicants, and a free Venn diagram generator (meta-chart.com) was used to visualize the data.

To analyze if women applicants more often than men work in traditional gender-segregated medical research areas such as pediatrics, gynecology, dermatology, or care sciences, the number of women and men MeSH terms (nodes) associating with the mentioned fields were counted using the MeSH Browser and related to total MesH term (node) number for either gender.

### Composite Bibliometric Score

The composite bibliometric score was derived by the KI library analytic team based on articles and reviews published from 1995 to the time of application and available in the Web of Science. KI librarians manually verified all published articles using the PMID, reported in the application, to ensure that the applicant's name was included in the article, with the correct author position. If the PMID had been incorrectly input by the applicant, the librarians matched the publications to the author by searching the journal, article title, and author. If the publication matched its PMID, but the author's name was missing, the full text of the publication was checked for the name. The score is adapted from a composite bibliometric score used previously (Holst and Hägg, 2018), inspired by Wennerås and Wold (1997) but consists of seven parts rather than six: (1) the number of publications, (2) the total number of citations to those publications, (3) the share of publications where the applicant was the first author, (4) the share of publications where the applicant was the last author, (5) the H index, (6) the share of publications in high impact journals within its field (field-normalization metric), and (7) a binary indicator for having any publication in a high impact journal overall. The sixth variable was included to represent perceived quality of the publication list. “Field,” as a bibliometric parameter, was defined using Clarivate's journal categorization. Within a field, the top two journals by Journal Impact Factor were considered high impact, and overall, the top 30 journals were considered high impact. Citations were retrieved from the Web of Science and Journal Impact Factors from Journal Citation Reports, both maintained by Clarivate Analytics. Each part of the score was log-transformed and normalized within the applicant pool so that the smallest value corresponds to a standardized value of 0 and the largest value to a standardized value of 1, leading to a ranking of applicants. The composite score is the sum of these seven standardized variables. The highest and lowest possible score is thus 7 and 0, respectively.

### Statistical Analysis

The composite score was calculated for the applicants selected for the external review step; hence, the standardization of the score was only done on the selected applicants. For analyses of pooled data (2014–2017 together), applicants who applied to the same position multiple times were identified, and only the first instance of application was included in the analyses. The total number of applicants included in separate analyses is 360 (207 applications for Assistant Professor and 153 for Senior Researcher). When repeat applicants are removed for pooled analysis of the full datasets for 2014–2017, keeping only the first instance of application, 186 unique applicants for Assistant Professor and 117 unique applicants for Senior Researcher were included, a total of 303 applicants.

Linear regression models were used to quantify the association between the composite bibliometric score (*x*-variable) and the external reviewer score (*y*-variable). In the full model, the analysis was performed adjusting for gender and including an interaction term to assess difference in slopes between men and women. In separate analyses, data were stratified by gender and year (including full data without removing duplicated individuals when each year was analyzed separately). In the results, the slope estimate addresses whether there is an association between composite bibliometric scores and external reviewer scores. The gender term addresses whether there is an overall difference in external reviewer scores for men and women. The interaction term addresses whether there is a difference between the slopes for men and women. The *R* value is the correlation coefficient revealing the strength of the association between composite bibliometric scores and external reviewer scores. The *R*-square is the proportion of variation of external reviewer scores that can be explained by the model. Df—degrees of freedom—is related to the number of samples, and *F* = *F*-statistic, whether the model is a good fit. *P*-values for estimates are considered statistically significant at *p* < 0.05. Analyses were performed in R version 3.6.0.

## Results

**Figure 1:**
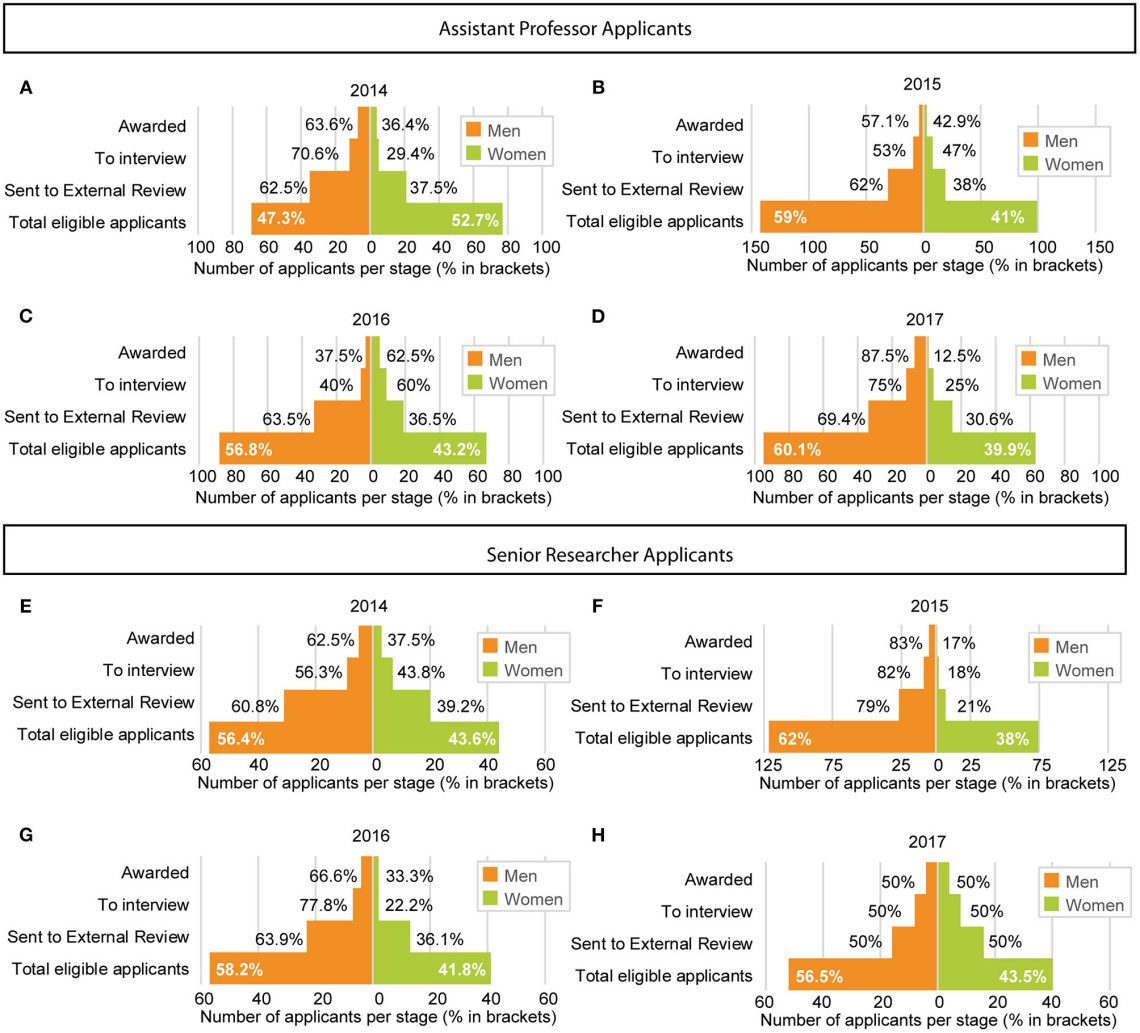
Number of applicants and proportions at each step of the recruitment process for the career ladder positions at Karolinska Institutet, divided by each year [**(A)** 2014, **(B)** 2015, **(C)** 2016, **(D)** 2017] for applications for assistant professor positions **(A–D)** or for Senior Researcher positions **(E–H)**. In five of the eight calls, the % of men awarded grants was higher than the % of men in the eligible applicant pool **(A, D–G)**.

Between 2014 and 2017 the KI Career Ladder program attracted 1,187 eligible applicants, of which 681 were men and 506 were women (Figure 1). Some applicants have applied multiple times over these years. Thirty-nine men were awarded a position, resulting in an overall success rate of 5.72% (success rate range: 2.8–10.1%), while 23 women were awarded a position, resulting in an overall success rate of 4.55% (success rate range 1.4–10%). Although men constitute 57% of the applicant pool, they constitute 63% of the awardees. Conversely, women constitute 43% of applicants but only 37% of the awardees. The characteristics of the applicants selected for external peer review, and analyzed in this manuscript, are listed in Table 1. In total, 360 applicants were selected for external peer review, and form the basis of our analyses here. Where applicants have applied over multiple years, only the first instance of application is included when grouped analyses, including several years, are performed. Overall, there are no statistically significant differences in external reviewer—or bibliometric scores between men and women. The number of men and women retained at each step, including eligible applicants, those selected for external peer review, those selected for interview, and those finally selected for funding/positions is depicted in Figure 1 and Table 1.

**Table 1:** Basic characteristics of the applicants.

		**2014**					**2015**					**2016**					**2017**				
		**Men**		**Women**		***p***	**Men**		**Women**		***p***	**Men**		**Women**		***p***	**Men**		**Women**		***p***
		***N* or**	**SD**	***N* or**	**SD**		***N* or**	**SD**	***N* or**	**SD**		***N* or**	**SD**	***N* or**	**SD**		***N* or**	**SD**	***N* or**	**SD**	
		**mean**		**mean**			**mean**		**mean**			**mean**		**mean**			**mean**		**mean**		
Assistant professor	*N*	35		21			31		19			33		19			34		15		
	Merit score [m (SD)]	4.62	0.79	4.40	0.46	ns	4.37	0.66	4.37	0.59	ns	4.49	0.70	4.77	0.76	ns	4.36	0.58	4.39	0.75	ns
	Bibliometric score	3.01	0.83	2.81	0.67	ns	3.09	0.62	3.11	0.97	ns	3.15	0.88	3.44	0.97	ns	2.93	0.92	3.17	0.92	ns
Senior researcher	*N*	31		20			27		7			23		13			16		16		
	Merit score [m (SD)]	4.67	0.59	4.61	0.80	ns	4.98	0.59	4.86	0.36	ns	4.69	0.55	4.38	0.75	ns	5.18	0.60	4.82	0.72	ns
	Bibliometric score	3.18	0.87	3.07	0.72	ns	3.60	0.73	3.78	0.65	ns	3.22	0.90	2.90	1.05	ns	2.94	0.90	2.66	1.12	ns

### No Evidence for Gender Segregation of Applicant Field

**Figure 2:**
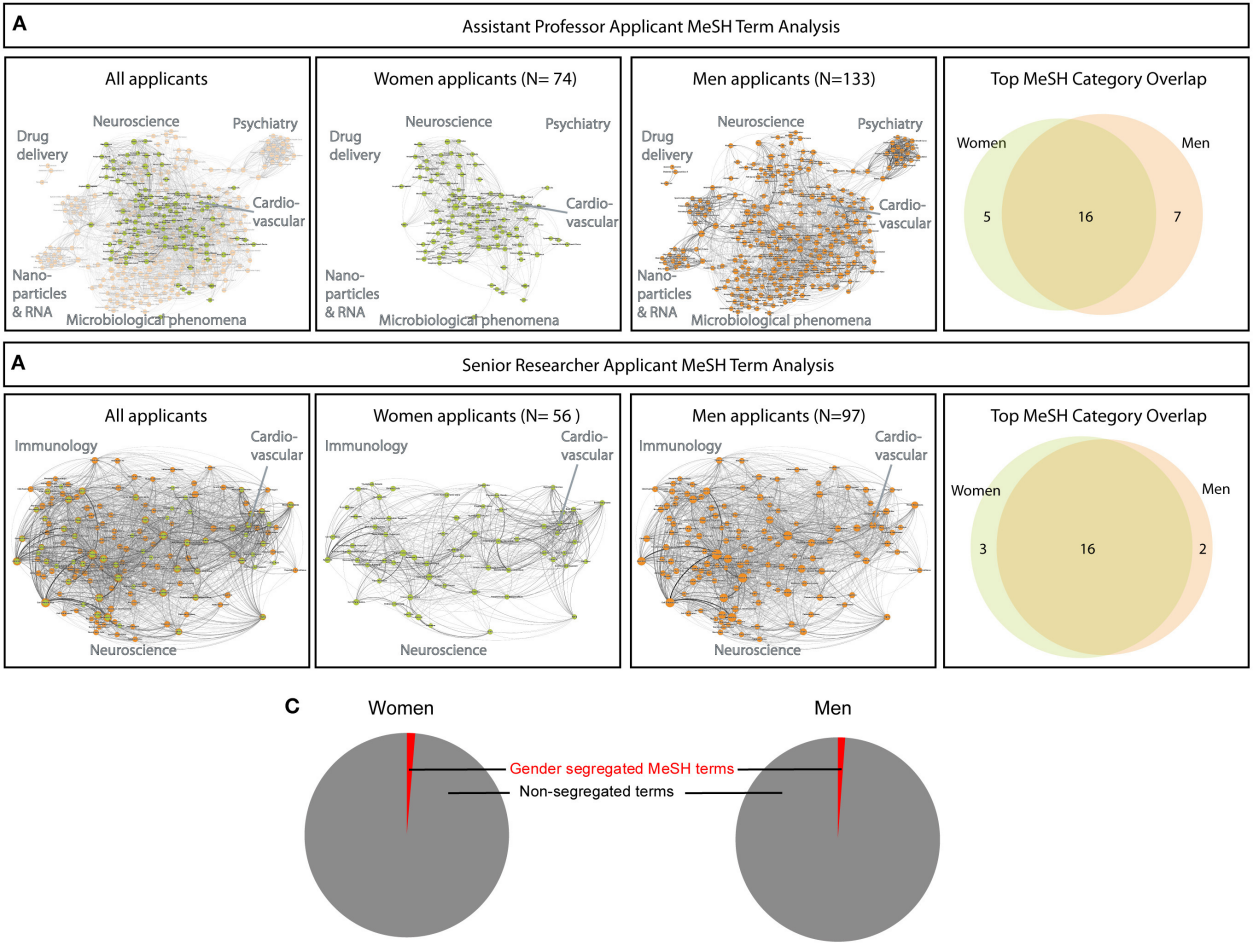
Analysis of field representation among men and women applicants using MeSH (medical subject headings) term analysis. **(A)** Applicants for assistant professor positions are more diverse and encompass 207 applicants (74 women and 133 men). Field representation is not obviously skewed by biased fields such as cardiovascular medicine, which is represented by MeSH terms among both men and women. There is a high degree of overlap in MeSH terms among men and women applicants as seen in the Venn diagram at right. **(B)** Men and women applicants for Senior Researcher positions were fewer in total (153), are highly similar, and show near-complete overlap in MeSH terms, as seen in the Venn diagram at the far right. **(C)** Analysis of MeSH terms for gender-segregated topics revealed a very small fraction of gender-segregated MeSH terms among men (1.2%) or women (1.3%) applicants.

The advertised positions are open to all fields of biomedical research, and as such, no discrimination per field should be present. However, a potential difference underlying unequal success rates for men and women could, nonetheless, be an unequal representation in different subfields of biomedical research. To determine field representation within the applicant pool with respect to gender, we extracted MeSH terms from the applicants' publication records and built one MeSH network each for men and women applicants, and assessed their overlap (Figure 2). Because there were almost twice as many men as women assessed for Assistant Professor positions, the MeSH network for men contains more terms and is larger (Figure 2A). There is a great degree of overlap between the MeSH networks for men and women, but men also contained unique clusters for Psychiatry and Psychology (typically a woman-enriched field), Drug Delivery, and Microbiological phenomena. Research in cardiovascular medicine, a field enriched in men, was present among MeSH terms for both the women (Coronary Disease and Acute Coronary Syndrome) and the men (Cardiovascular Disease). Similarly, Neuroscience was also present among the MeSH terms for both the women and men. In the pool of applicants for Senior Researchers, there was a near-total overlap in MeSH terms (Figure 2B), though again, there were nearly twice as many men as women selected for evaluation. The percentage of gender-segregated MeSH terms was low, with 1.2% among men and 1.3% among women applicants (Figure 2C). In sum, the pool of applicants does not appear biased based on overrepresentation of sex-segregated fields of medicine/research and show a high degree of field similarity, in particular, among the Senior Researcher applicants.

### Women Receive Lower Merit Scores for Equal Objective Merits

To test whether men and women applicants are similarly ranked for merits based on their track records, we tested whether their external reviewer scores were correlated with an objective measure of their productivity: the composite bibliometric score, which factors in the number of publications, citations, share of first authorships, share of last authorships, the H index, share of high-impact publications, and whether they have any high impact publication overall (Table 2). This score was adapted from previous work (Wennerås and Wold, 1997; Holst and Hägg, 2018) and is based on parameters chosen to reflect scientific output, impact, ability to lead a research project at a junior (post doc) or senior (group leader) level, consistency of scientific impact, visibility of produced research, and ability to publish in high-ranking journals.

**Table 2:** Composite score components and relevance.

	**Composite score component**	
1	Number of publications	Output
2	Total number of citations	Impact
3	Share of publications in which the applicant was the first author	Ability to lead a project (Junior)
4	Share of publications in which the applicant was the last author	Ability to lead a project (Senior)
5	H index	Consistency of impact across publications
6	Share of publications in high impact journals within its field	Visibility of research and likely impact within its field
7	Binary indicator for having any publication in a high impact journal overall	Bonus for high-ranking publication

**Figure 3:**
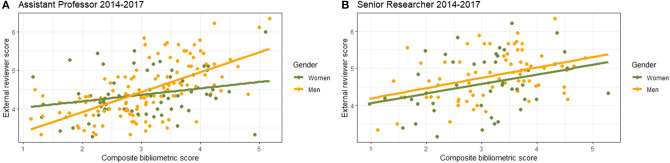
Linear regression associations between composite bibliometric scores and external reviewer scores received on merits combined for all years (2014–2017) and stratified by gender for applications for **(A)** Assistant Professor and **(B)** Senior Researcher positions.

Overall, associations were positive and statistically significant between composite bibliometric scores and external reviewer scores for both Assistant Professor positions (slope = 0.167, *P*-value = 0.034) and Senior Researcher positions (slope = 0.257, *P*-value = 0.012, Figure 3, Table 3A). The model had a better fit for the Assistant Professor applicants, compared with the Senior Researcher applicants, while the latter had a steeper slope. For the Assistant Professor applicants, men had a lower external reviewer score at intercept (est.= −0.97, *P*-value = 0.0022), but a significant interaction showed that men had a steeper slope (greater increase in awarded reviewer scores for equal merits) than women. Thus, at the highest composite scores, men received higher external reviewer scores than women in the Assistant Professor category (est. = 0.349, *P*-value = 0.0005). Indeed, stratifications on gender showed that the slope difference was evident for men and women applying for Assistant Professor positions (Table 3B, Figure 3). However, neither the gender effect alone, nor the interaction term, was statistically significant for Senior Researcher applicants. In summary, for each additional point in composite bibliometric score, women applying for Assistant Professor positions receive only 32% of the external reviewer score that men receive, and women applying for Senior Researcher positions receive 92% of the score men receive.

**Table 3A:** Linear regression results for associations between composite bibliometric scores and external reviewer scores in independent individuals (re-applying applicants are included only for their first application).

	**Assistant professor 2014–2017**							**Senior researcher 2014–2017**					
	**Est**	***P*-value**	***R***	***R*-square**	**df**	***F***		**Est**.	***P*-value**	***R***	***R*-square**	**df**	***F***
Slope	0.167	0.034	0.55	0.30	182	25.9	Slope	0.257	0.012	0.39	0.15	113	6.58
Gender (men)	−0.970	0.0022					Gender (men)	0.104	0.81				
Interact	0.349	0.0005					Interact	0.021	0.87				

**Table 3B:** Linear regression results for associations between composite bibliometric scores and external reviewer scores in independent individuals (re-applying applicants are included only for their first application), stratified by gender.

	**Assistant professor 2014–2017**						**Senior researcher 2014–2017**					
	**Slope**	***R***	***R*-square**	**df**	***F***	***P*-value**	**Slope**	***R***	***R*-square**	**df**	***F***	***P*-value**
Men	0.516	0.61	0.38	121	72.8	5.0E−14	0.279	0.38	0.14	70	11.8	0.001
Women	0.167	0.26	0.07	61	4.44	0.039	0.257	0.32	0.11	43	5.04	0.029

We next stratified the analyses by application year (and position), now including all applicants in the data (repeat applicants included where applicable). The results showed a gender difference in Assistant Professor assessments in the years 2014–2015, but no associations were found between composite bibliometric scores and external reviewer scores. In contrast, for 2016–2017, the associations were apparent, but gender was not significant (Supplementary Table 1, and Supplementary Figure 1). The null significance for slope is driven by the null correlation between composite scores and external reviewer scores in women for the years 2014–2015. For the Senior Researcher positions, the effects are not statistically significant in stratifications, probably due to lower power when splitting the data.

There are fewer data points among Senior Researchers than Assistant Professors, but it is noteworthy that the model fit is worse in the Senior Research model than in the Assistant Professor model (*F* = 6.58 in the Senior Researcher model vs. *F* = 25.9), which may also suggest that other merits than publications (e.g., prizes, invitations to speak, editorial work) may impact on reviewer scores later in the career. Finally, it is worth noting that the two Senior Researcher applicants with the highest bibliometric scores were women (composite bibliometric scores 5.28 and 5.16), who were nevertheless given merit scores lower than the third and fourth applicants, who were men (composite bibliometric scores 5.04 and 4.86). The women received 4.33 and 4.72 as merit scores, while the third and fourth applicants received a merit score of 5.56 and 5.28, reinforcing the conclusion that bias may have the strongest impact at the top of the competition.

## Discussion

**Figure 4:**
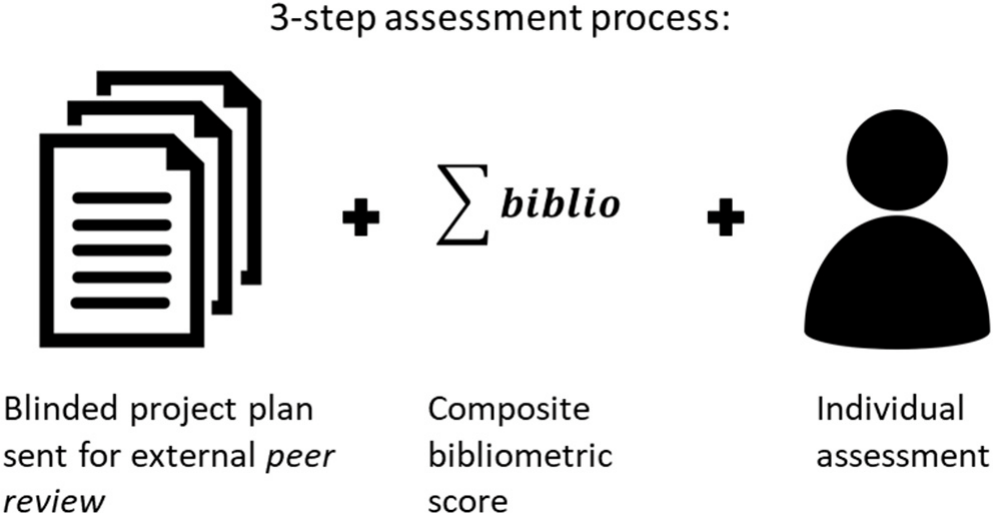
Proposed three-step review process to minimize impact of bias. Project plans can be reviewed blinded by reviewers when applicants are junior, reducing risk of bias. A composite bibliometric score is calculated to support assessment of past productivity and impact, reducing bias (some metrics have been shown to be unfairly biased by gender). An individual assessment, performed by a peer reviewer, integrates scores from the project plan and the composite bibliometric score, to assess the feasibility of the project. Together, these three steps reduce bias while allowing for an assessment of a project's innovativeness and an applicant's competence to execute the project.

In this paper, we asked whether men and women are ranked equally when presenting equal merits. We analyzed gender distributions across a three-step recruitment process for group leader positions at the Karolinska Institutet in Sweden (Assistant Professor and Senior Researcher) over a period of 4 years. We demonstrated clear differences comparing objective quantified productivity and external reviewer scores for merits between men and women, in which women, on average, received lower merit scores for equal bibliometric achievements (as illustrated in Table 3, Figures 3, 4). Thus, gender bias in recruitments to higher academic positions is likely to continue to reinforce the unbalanced numbers in professorships in Sweden.

Our data stand in contrast to some recent reports that women are favored in STEM hiring decisions (Williams and Ceci, 2015) or are considered more competent and intelligent (Eagly et al., 2020). However, these studies addressed overt stated preference, in which individuals may overcompensate to avoid the risk of presenting bias. Preference for either gender (ignoring merits) is not desirable and could lead to a backlash in the community. The current study analyzed a real-world peer review procedure, in which the reviewers did not know that their assessments would be analyzed *post hoc*, revealing gendered assessments of applicant merits. Our study did not show an overall difference in the scores for men and women (Table 1), which could be taken to mean that men and women are scored equally and fairly. However, we regressed composite bibliometric scores with merit scores and show that men are awarded higher scores for equal composite bibliometry merits (Table 3, Figures 3, 4). Based on the composite bibliometric score, 20 men and 14 women (59 vs. 41% of final top applicants) were top ranked in the calls for Assistant Professor. However, based on external reviewers' merit scores, 23 men and 11 women (68 vs. 32% of final top applicants) were top ranked. These numbers are too small to test statistically with confidence, but statistically significant differences in how men and women are assessed (Table 3A) resulted in widening the gap from 6 more men, to 12 more men. Although composite bibliometry is also associated with limitations, it may present a more rigorous approach to assess candidates based on their merits, with less gender bias.

When assessing the data for Assistant Professors there is a striking difference in the peer review process comparing 2014–2015 with 2016–2017 because gender differences are apparent in the first years but not in the latter (Figure 3, Supplementary Table 1). However, there was no change in the instructions sent out to external reviewers (Appendix) that could explain this deviation. There were no instructions concerning gender bias, nor suggestions as to how to deal with bias. In light of the data presented here, and general awareness of the challenges, KI has started to talk about bias (https://staff.ki.se/assessment-bias-and-career) and has launched a web training program for all reviewers. This effort is a step in the right direction, hopefully with more to come.

A limitation in the current study is the small sample number (number of applicants) and the wide array of research fields in the group of applicants. We show in Figure 2 that the men and women applicants generally represent overlapping subfields of research, but this was not possible to correct for in the linear regressions in Figure 3, since the datasets in Figures 2, 3 were maintained separately to ensure anonymity for the described applicants. Large-scale studies, perhaps of national grant applications, correlating merit scores and composite bibliometric scores, could more thoroughly address how research field impacts on applicant scoring. Another limitation of the study is that replication would have been desired, but unfortunately, we cannot repeat the study in a different European country since other universities do not use the same process as ours, not even within Sweden. Other countries do not have open access to information either, which is guaranteed by law in Sweden. Nevertheless, the composite bibliometric score correlated well with merit scores awarded to men, and not so well with scores awarded to women, suggesting that the peer review of men and women is either biased or based on different grounds.

Fortunately, there is much data available offering guidance for the construction of a quality-controlled peer-review process. Peer reviewers for NIH applicants show low agreement on the same application (Pier et al., 2020), and a recent preprint in PsyArXiv concluded that at least 12 reviewers per application are needed in order to obtain reliable scores (Forscher et al., 2019). Finland has launched a program in which governmental agencies join forces to establish a standardized template, to be used across all funding bodies, for evaluating researchers in a fair and equal way (https://avointiede.fi/sites/avointiede.fi/files/Vastuullinen-arviointi-luonnos_1.pdf), which may also standardize procedures and reduce the impact of bias. Based on the bias we identified here, and previous exhaustive work showing biased review processes (Wennerås and Wold, 1997; Moss-Racusin et al., 2012; Holst and Hägg, 2018; Witteman et al., 2019), we propose a three-step semi-blinded review process (Figure 4). We suggest that project proposals should be assessed blinded to applicant gender, merits could be quantified using the composite bibliometric score, and a reviewer would integrate these two components into an overall assessment. Whether this procedure is better at identifying top-qualified individuals should be further researched, for example, through a field experiment. It is important to note that while we consider merit quantification to be a possible improvement over current practice, bibliometry itself is biased as well (Caplar et al., 2017) and may contribute to continued discrepancies. Furthermore, the suitability of assessing individuals based on publication metrics is a heavily debated issue. However, considering the challenges in fairly assessing women, composite bibliometry is an improved measure of productivity compared with reviewer assessment, which we show is biased. Reviewers find citation scores and the number or proportion of papers in the most highly cited percentage most useful for assessing candidates (Gunashekar et al., 2017), and future work to further optimize the composite bibliometric score for assessing candidates should aim to ensure that in-built citation bias does not compound existing bias with metrics that appear deceptively unbiased. To address citation gender bias, the composite bibliometric score could also be corrected by a field-specific “bias factor.” Finally, we also propose that granting bodies should self-assess and quality-control their peer-review procedures by testing whether applicants receive merit/competence scores that correlate with their productivity and whether scoring is well matched for women and men applicants.

The difference in success rates for men and women vary widely across position and year, but is higher for men in general. Furthermore, in individual years, the proportion of men or women in the recruitment process is not maintained throughout individual steps, and the proportion of women tends to decrease at each consecutive step (Figure 1), an expected result if bias is greatest at the top of the competition (Figure 3). If women constitute 53% of the eligible applicant pool, one would expect 53% of the positions to be awarded to women. Local variation is expected, but consistently higher success rates for men throughout the assessment process suggest men are consistently better, which is not supported by our data. Our analysis using the composite score clearly shows that men are more highly rewarded for equal merits (Table 3). There is a fundamental flaw in the “meritocracy” when demographic groups are eliminated as a consequence of not reflecting the norm in academia (Leslie et al., 2015).

Sweden is one of the most gender equal countries in Europe (European Institute for Gender Equality, 2017), yet only 25–28% of professors are women (Swedish Higher Education Authority, 2018; European Commission, 2019). Comparing the percentages of women professors, Sweden is in the 13th place, and instead Bulgaria and Latvia lead with 45–54% women professors (“Grade A” positions) (European Commission, 2019; Hovdhaugen and Gunnes, 2019). In the 1950s in Sweden, men and women were equally highly educated, but since the 1960s, more women than men have been highly educated with a difference of six percentage points. Currently, 39% of women born in 1976 were highly educated at the age of 40 years, while only 23% of men were highly educated. Between 46 and 49% of doctoral students in Sweden are women (Swedish Higher Education Authority, 2018). In 2017, within medical and health sciences, agricultural and veterinary sciences and social sciences: 59% of doctoral degrees were awarded to women and 41% to men. Thus, education levels cannot explain the dearth of women professors in Swedish universities. Using data from the Swedish Research Council (known as Swedish Medical Research Council at the time), Wennerås and Wold showed in 1997 that women applicants for a postdoctoral fellowship had to be 2.5 times more productive than men to be considered equally merited (Wennerås and Wold, 1997). A replication study in 2008 did not replicate the findings of gender bias, but showed that highly merited women were not rewarded with scores as high as men for equal numbers of citations or high impact papers (Sandström and Hällsten, 2008) and correction (Sandström and Hällsten, 2020). Since then, numerous studies worldwide have demonstrated bias in almost all processes impacting on career progression including hireability of men vs. women with identical merits (Steinpreis et al., 1999), desire to mentor men vs. women with identical merits (Moss-Racusin et al., 2012), and probability of citation of similar papers authored by men or women (Moss-Racusin et al., 2012; Caplar et al., 2017). Over 20 years later, an analysis by one of the authors (SH) showed that bias against women and non-European men is still significant in the recruitment of junior faculty (Assistant Professors^1^) to Principal Investigator positions at KI (Holst and Hägg, 2018), the results of which we confirm and extend here. Although awareness of bias is increasing, and processes are being implemented to prevent bias from impacting negatively on career progression, recent data show that women are still not chosen when intellectual ability is being sought after (Bian et al., 2018). Our data similarly suggest that bias may be more prevalent in the highest tier of competition for academic positions in biomedical research, slowing the achievement of parity in professorships in the academia.

To conclude, in order to attract and maintain the best scientists in the academic career track, all individuals should have equal opportunities and be reviewed in transparent systems on equal terms. Ensuring a meritocracy with the best individuals in the academia will require more work to ensure quality-controlled recruitment and assessment procedures.

## Data Availability Statement

Publicly available datasets were analyzed in this study. The data are publicly available and can be requested from the Registrar at Karolinska Institutet, Sweden.

## Author Contributions

EA conceptualized the study, conducted the data curation, performed the formal analysis, conducted the investigation, developed the methodology, was in charge of the project administration and visualization, prepared and wrote the original draft, and wrote, reviewed, and edited the article. CH performed the MesH term analysis and wrote, reviewed, and edited the article. SH conceptualized the study, conducted the data curation, performed the formal analysis, conducted the investigation, developed the methodology, was in charge of the project administration and visualization, prepared and wrote the original draft, and wrote, reviewed, and edited the article.

## Conflict of Interest

EA, CH, and SH have all obtained positions/funding within the KI Career ladder described here.

## Footnotes

^1^Assistant Professor in the KI Career Track 2014–2018 was a 4-year position as independent group leader/PI, typically the first faculty appointment. Senior Researcher was a 5-year position, typically awarded after completion of an Assistant Professor position, the equivalent of Associate Professor.

## Supplementary Material

Supplementary Figure 1 Linear regression associations between composite bibliometric scores and external reviewer scores received on merits, stratified by gender, and divided by each year for applications to Assistant Professor positions **(A-D)** and for applications to Senior Researcher positions **(E-H)**. Model outputs are found in Supplementary Table 1.

Supplementary Table 1 Association between composite bibliometric scores and external reviewer scores by year and position.
