# Supplementary figures and images for: Gender Bias Impacts Top-Merited Candidates
Source: Front Res Metr Anal. 2021 May 10;6:594424. doi: 10.3389/frma.2021.594424 (PMC8141636; doi:10.3389/frma.2021.594424)

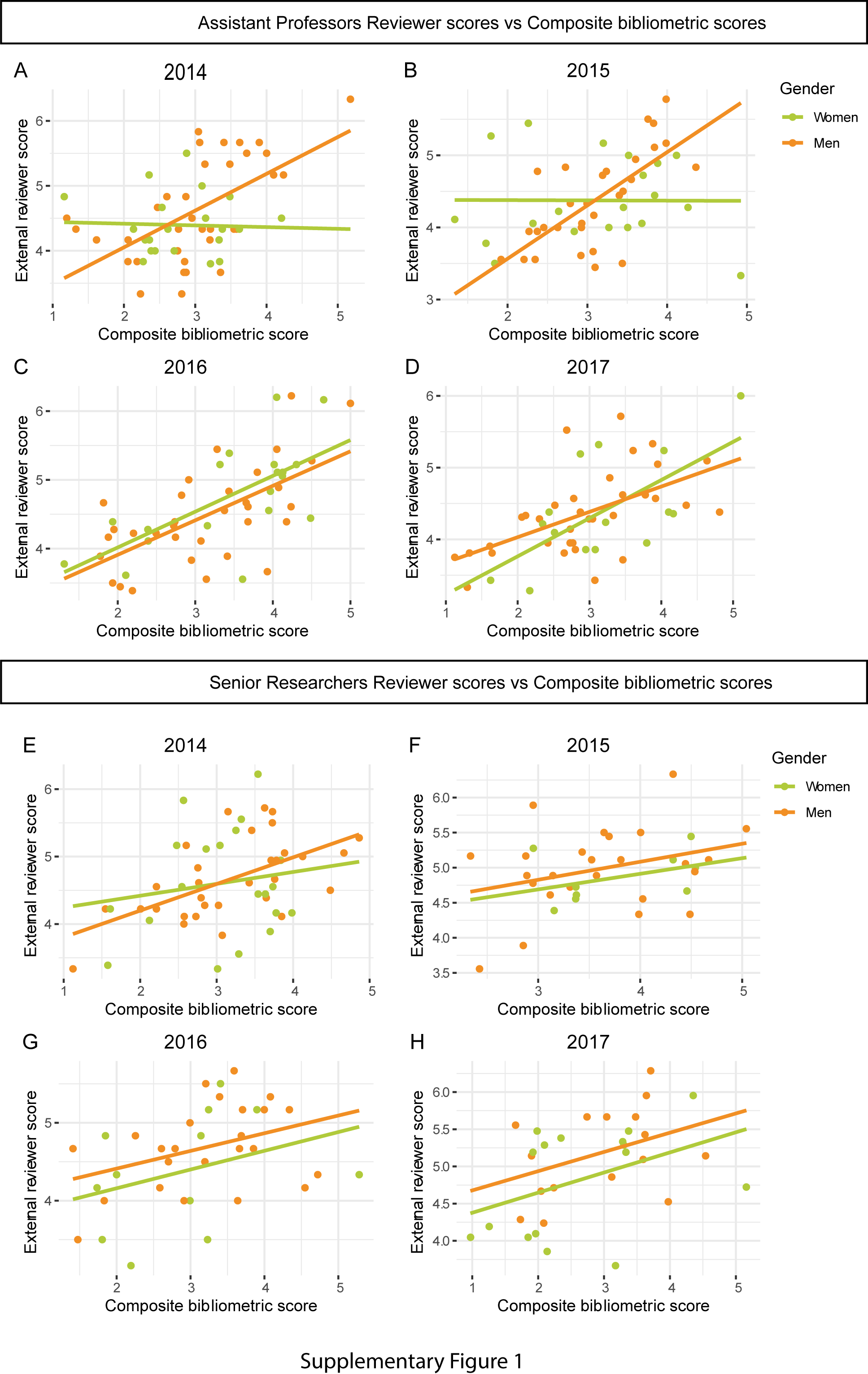

Supplement: Supplementary Figure 1 — Linear regression associations between composite bibliometric scores and external reviewer scores received on merits, stratified by gender, and divided by each year for applications to Assistant Professor positions (A-D) and for applications to Senior Researcher positions (E-H). Model outputs are found in Supplementary Table 1. [file Image_1.TIF]
