# Supplementary material for: Gender Bias Impacts Top-Merited Candidates
Source: Front Res Metr Anal. 2021 May 10;6:594424. doi: 10.3389/frma.2021.594424 (PMC8141636; doi:10.3389/frma.2021.594424)
Supplement: Supplementary Table 1 — Association between composite bibliometric scores and external reviewer scores by year and position. [file Table_1.docx]

Supplementary table 1. Association between composite bibliometric scores and external reviewer scores by year and position.

|  | **Assistant Professor 2014** | | | |  |  |  | **Senior Researcher 2014** | | | |  |  |
| --- | --- | --- | --- | --- | --- | --- | --- | --- | --- | --- | --- | --- | --- |
|  | **Estimate** | **P-value** | **R** | **R-square** | **df** | **F** |  | **Estimate** | **P-value** | **R** | **R-square** | **df** | **F** |
| **Slope** | -0.02625 | 0.8956 | 0,547723 | 0.3069 | 52 | 7.67 | **Slope** | 0.1761 | 0.387 | 0,413884 | 0.1713 | 47 | 3.239 |
| **Sex (Men)** | -1.54913 | 0.0291 |  |  |  |  | **Sex (Men)** | -0.6584 | 0.398 |  |  |  |  |
| **Interaction** | 0.59240 | 0.0145 |  |  |  |  | **Interaction** | 0.2188 | 0.369 |  |  |  |  |
|  | **Assistant Professor 2015** | | | |  |  |  | **Senior Researcher 2015** | | | |  |  |
|  | **Estimate** | **P-value** | **R** | **R-square** | **df** | **F** |  | **Estimate** | **P-value** | **R** | **R-square** | **df** | **F** |
| **Slope** | -0.00329 | 0.979367 | 0,556776 | 0.3119 | 46 | 6.95 | **Slope** | 0.22278 | 0.443507 | 0,338674 | 0.1147 | 30 | 1.295 |
| **Sex (Men)** | -2.29545 | 0.001023 |  |  |  |  | **Sex (Men)** | 0.03557 | 0.977093 |  |  |  |  |
| **Interaction** | 0.74182 | 0.000748 |  |  |  |  | **Interaction** | 0.03405 | 0.916863 |  |  |  |  |
|  | **Assistant Professor 2016** | | | | | |  | **Senior Researcher 2016** | | | | | |
|  | **Estimate** | **P-value** | **R** | **R-square** | **df** | **F** |  | **Estimate** | **P-value** | **R** | **R-square** | **df** | **F** |
| **Slope** | 0.52086 | 0.000421 | 0,660151 | 0.4358 | 48 | 12.36 | **Slope** | 0.24040 | 0.168 | 0,421307 | 0.1775 | 32 | 2.302 |
| **Sex (Men)** | -0.06912 | 0.910823 |  |  |  |  | **Sex (Men)** | 0.27969 | 0.691 |  |  |  |  |
| **Interaction** | -0.01894 | 0.915687 |  |  |  |  | **Interaction** | -0.01356 | 0.951 |  |  |  |  |
|  | **Assistant Professor 2017** | | | | | |  | **Senior Researcher 2017** | | | | | |
|  | **Estimate** | **P-value** | **R** | **R-square** | **df** | **F** |  | **Estimate** | **P-value** | **R** | **R-square** | **df** | **F** |
| **Slope** | 0.5319 | 0.000903 | 0,603407 | 0.3641 | 45 | 8.587 | **Slope** | 0.27046 | 0.0727 | 0,473709 | 0.2244 | 28 | 2,701 |
| **Sex (Men)** | 0.6249 | 0.284005 |  |  |  |  | **Sex (Men)** | 0.31235 | 0.6552 |  |  |  |  |
| **Interaction** | -0.1790 | 0.321711 |  |  |  |  | **Interaction** | -0.01178 | 0.9597 |  |  |  |  |
